# Therapeutic Potential of Selenium as a Component of Preservation Solutions for Kidney Transplantation

**DOI:** 10.3390/molecules25163592

**Published:** 2020-08-07

**Authors:** Aneta Ostróżka-Cieślik, Barbara Dolińska, Florian Ryszka

**Affiliations:** 1 Department of Pharmaceutical Technology, Faculty of Pharmaceutical Sciences in Sosnowiec, Medical University of Silesia, Kasztanowa 3, 41-200 Sosnowiec, Poland; bdolinska@sum.edu.pl; 2 “Biochefa” Pharmaceutical Research and Production Plant, Kasztanowa 3, 41-200 Sosnowiec, Poland; f.ryszka@biochefa.pl

**Keywords:** selenium, prolactin, organ preservation solution, Biolasol^®^, kidney transplantation

## Abstract

Selenium has strong antioxidant properties and diverse effects on the immune system. The aim of the study was to analyse the protective effect of selenium as a component of a kidney preservation solution on the prevention of ischemia-reperfusion injury of nephrons. The solution was modified by the addition of Se (1 µg/L), prolactin (0.1 µg/L) and Se with prolactin (1 µg/L Se + 0.1 µg/L PRL). The study used a model for storing isolated porcine kidneys in Biolasol^®^ (modified Biolasol^®^), which minimizes ischemia-reperfusion injury of grafts. The introduction of Se^4+^ ions at a dose of 1 µg/L into the Biolasol^®^ preservation solution in the form of Na_2_SeO_3_ caused an increase in the activity/concentration of the analysed biochemical parameters: aspartate transaminase, alanine transaminase, urea and protein. This suggests an adverse effect of Se^4+^ on nephron function during ischemia-reperfusion. The best graft protection was obtained by using Biolasol^®^ modified with the addition of selenium (IV) at a dose of 1 µg/L and prolactin at a concentration of 0.1 µg/L. We proposed the mechanism of prolactin action in the metabolic reduction of selenite (SO_3_^2−^) during ischemia/reperfusion.

## 1. Introduction

Selenium is a necessary bioelement for the proper functioning of the body. Its content in the adult human body is 10–20 mg and decreases in organs in the following order: liver (30%) = muscle (30%) > kidney (15%) > plasma (10%) >other organs (15%) [1,2]. It is estimated that the normal serum Se concentration should be 60–120 ng/mL [3]. The daily selenium intake (RDA) recommended by the World Health Organization (WHO) is 55 µg/day [4]. In Poland, it is 40 µg/day and 65 µg/day for women and men, respectively. Selenocysteine, selenomethionine, selenoneine, selenite and selenate show high bioavailability of selenium. Se ions in inorganic compounds are absorbed in 84% and reduced during absorption by means of reduced glutathione in the presence of NADPH (nicotinamide adenine dinucleotide phosphate). The half-life of selenium in the human body is 65–115 days. Selenium is mainly incorporated in amino acids (Se-Met, selenomethionine; Se-Cys, selenocysteine), which are present in almost every living cell. Selenium-dependent enzymes include glutathione peroxidases (GPX1-GPX4 and GPX6), thioredoxin reductases (TrXNRD1-TrXNRD3), methionine sulfoxide reductase (MSRB1) as well as type I and III iodothyronine deionidases (DIOs). In addition, the human body contains selenoproteins (SelenoW, SelenoH, SelenoT, SelenoP, SelenoM, SelenoN, SelenoK, SelenoS, SelenoV, SelenoI, SelenoO), which perform various functions, including proper protein folding and participating in the endoplasmic reticulum stress response. GPX plays an important role in antioxidant cell defence. It catalyses the reduction of organic hydroperoxides (R-O-O-R’) and hydrogen peroxide (H_2_O_2_) using reduced glutathione as an electron donor. In addition, it inhibits the lipid peroxidation process. The level of selenium in the body is quite often determined indirectly by measuring the activity of glutathione peroxidase. DIOs regulate thyroid hormone activity. They convert thyroxine (T_4_) to 3,5,3′-triiodothyronine (T_3_). TrXNRD is involved in the regulation of redox potential, cell proliferation (affecting the process of apoptosis) and immune response [2,5,6,7,8].

Selenium has strong antioxidant properties and diverse effects on the immune system. It affects the maintenance of oxidoreductive balance of cells. It regulates redox homeostasis as part of intracellular antioxidative systems with catalase (CAT), superoxide dismutase (SOD), glutathione (GSH), vitamin E, carotenoids and ascorbic acid. It participates in maintaining the integrity of cell membranes and regulates prostacyclin biosynthesis [5]. In high concentrations, it has immunosuppressive effects, in low concentrations, it has immunostimulatory effects. It affects non-specific macrophages (humoral response) and B and T lymphocytes (cellular response). Selenium deficiency reduces T cell proliferation and NK (natural killer) cell activity [3]. Selenium is involved in the regulation of pro-inflammatory gene and cytokine expression. It reduces the expression of COX-2 (cyclooxygenase-2), iNOS (inducible nitric oxide synthase) and TNFα (tumour necrosis factor α). It inhibits the expression of the transcription factor NF-κB (nuclear factor-κB) and IRF3 (IFN-regulatory factor 3) induced by TLR3 (Toll-like receptor 3) or TLR4 (Toll-like receptor 4) agonists [9,10].

It is suggested that selenium has multidirectional effects on the human body. In recent years, there have been numerous literature reports indicating the possibility of its multidirectional therapeutic effect. It has been confirmed that it can have antioxidant, anti-inflammatory, antimutagenic, antitumoral or chemopreventive, antiviral, antibacterial, antifungal and antiparasitic properties. It can also be used to prevent dementia and cognitive impairment [11]. It has been found that the increase in the acute inflammatory response correlates with the decrease in the concentration of bioelements, including selenium, especially when the concentration of C-reactive protein (CRP) rises above 80 mg/L [12]. This may explain the low selenium concentration in patients with organ failure [13].

**Figure 1:**
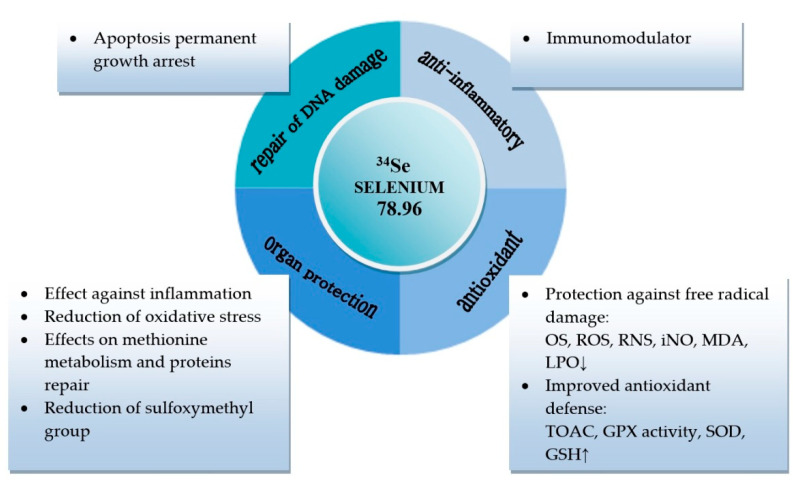
Role of selenium in organ function. OS, oxidative stress; ROS, reactive oxygen species; RNS, reactive nitrogen species; iNO, inhaled nitric oxide; MDA, malondialdehyde; LPO, lipid peroxidation; SOD, superoxide dismutase; TAOC, total antioxidant capacity; GPX; glutathione peroxidase; GSH, glutathione.

Toufektsian et al. conducted a study on the effect of a diet with reduced selenium content on cardiac function during ischemia-reperfusion in an isolated rat heart model. The authors found that GPx activity in the cytosolic and mitochondrial fraction decreased, and the severity of histopathological changes in myocardium increased compared to the control group [14]. In turn, Poltronieri et al. found that giving rats a diet containing sodium selenite at a dose of 6 µg Se/day improved post-ischemic myocardial regeneration. An increase in glutathione peroxidase activity in all tissues and a reduction in cardiac keratin kinase release were observed [15]. In turn, giving rats a diet containing 1.5 mg Se (sodium selenate) /kg body weight reduced arrhythmia induced by cardiac reperfusion [16]. Zapletal et al. studied the effect of selenium on liver function in the model of partial warm liver ischemia in the rat. Sodium selenite was administered to rats intravenously at a dose of 0.125 µg/g, 0.25 µg/g and 0.375 µg/g. An improvement in hepatic microcirculation was observed. ALT activity decreased, MDA (malondialdehyde) concentration remained unchanged. Total and reduced glutathione concentration increased [17]. Hasanvand et al. investigated the effect of selenium on renal IR (ischaemia-reperfusion) injury. Sprague-Dawley rats were given selenium (sodium selenate) intraperitoneally at a dose of 0.2 mg/kg. It was found that the bioelement reduced the concentration of MDA and urea in blood serum, whereas the activity of reduced glutathione (GSH) and superoxide dismutase (SOD) increased. Histopathological examination revealed less damage compared to the control group [18]. Ahmadvand et al. confirmed that intraperitoneal administration of Se at a dose of 1 mg/kg to rats improved renal function markers during ischemia-reperfusion. Selenium reduced serum creatinine, urea and MDA concentrations. GPx, GSH and SOD activity increased [19]. Based on the above studies, it can be concluded that selenium has antioxidant activity, protects nephrons, hepatocytes and cardiomyocytes, and prevents apoptosis. The effects of selenium on organ function [1,14,15,16,17,18,19,20] are shown in Figure 1.

Research results to date suggest that selenium may potentially have a therapeutic effect as a component of preservation solutions for kidney transplantation. The aim of the study was to analyse the protective effect of selenium, as a component of Biolasol^®^, on the prevention of ischemia-reperfusion injury of nephrons. The solution was modified by the addition of Se at a dose of 1 µg/L, prolactin at a dose of 0.1 µg/L and Se with prolactin (1 µg/L Se + 0.1 µg/L PRL). The addition of prolactin to Biolasol^®^ was dictated by the results of our previous studies. Prolactin lowers nephron damage markers during preservation and reperfusion of isolated porcine kidneys. It has antioxidant effects, which indicates the possibility of synergistic action of Se and PRL [21,22]. The study used a model for storing isolated porcine kidneys in Biolasol^®^, which minimizes ischemia-reperfusion injury of grafts. Its composition (anti-inflammatory agents, antioxidants, buffers, colloids, ROS scavengers, electrolyte) provides an optimal environment for organ preservation in the period between its explantation from the donor and implantation into the recipient [23,24,25,26].

## 2. Results

**Figure 2:**
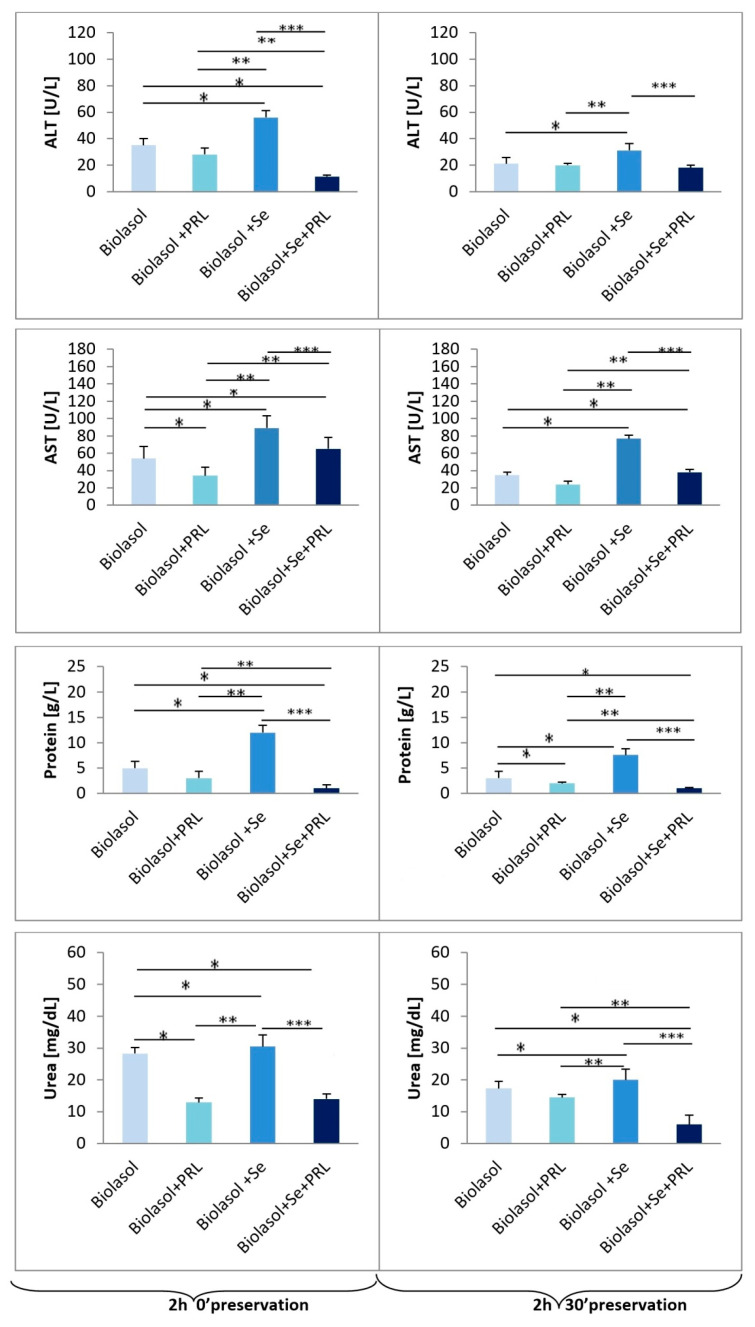
The effect of selenium on ALT, AST, protein, urea levels in porcine kidneys (2 h 0′and 2 h 30′ preservation). Means ± standard deviations (SD) are shown. The symbols represent a significant difference (*p* < 0.05) compared with the Biolasol^®^ group (*), the Biolasol^®^+PRL group (**), and the Biolasol^®^+Se group (***).

**Figure 3:**
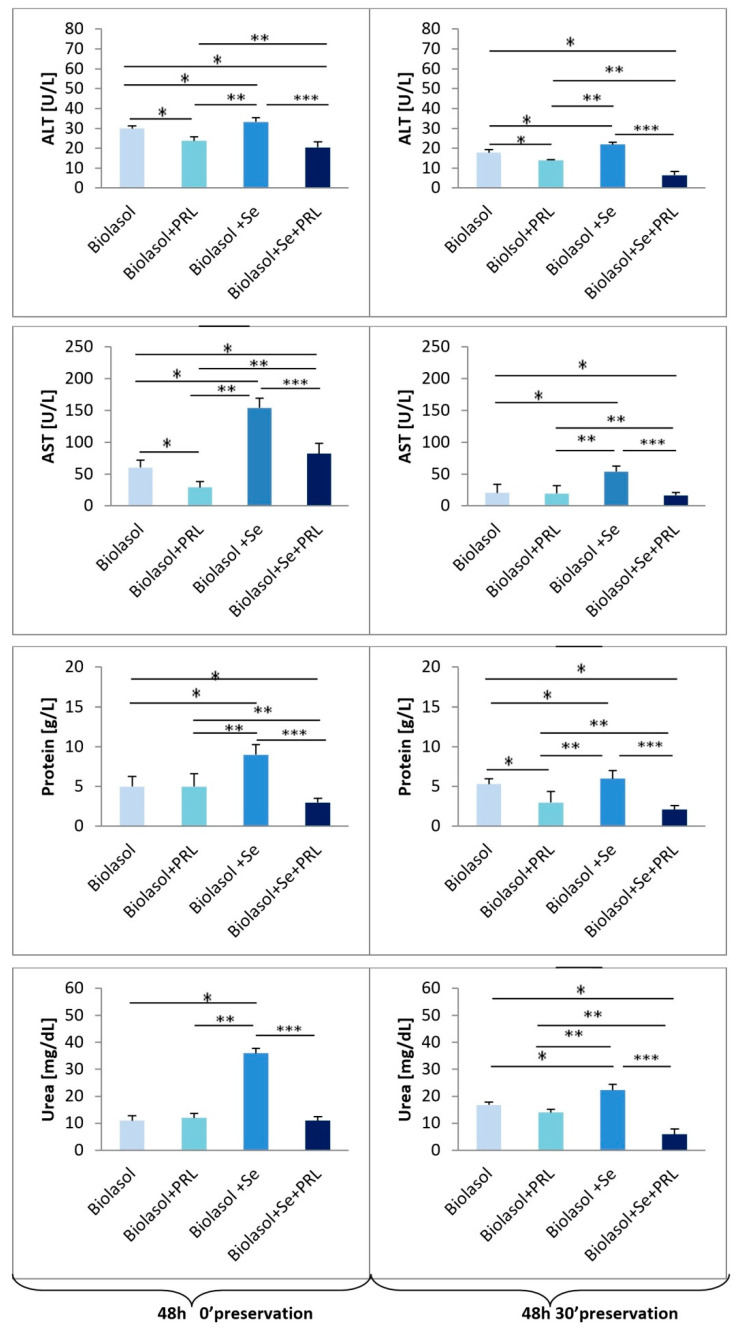
The effect of selenium on ALT, AST, protein, urea levels in porcine kidneys (48 h 0′and 48 h 30′ preservation). Means ± standard deviations (SD) are shown. The symbols represent a significant difference (*p* < 0.05) compared with the Biolasol^®^ group (*), the Biolasol^®^+PRL group (**), and the Biolasol^®^+Se group (***).

**Figure 4:**
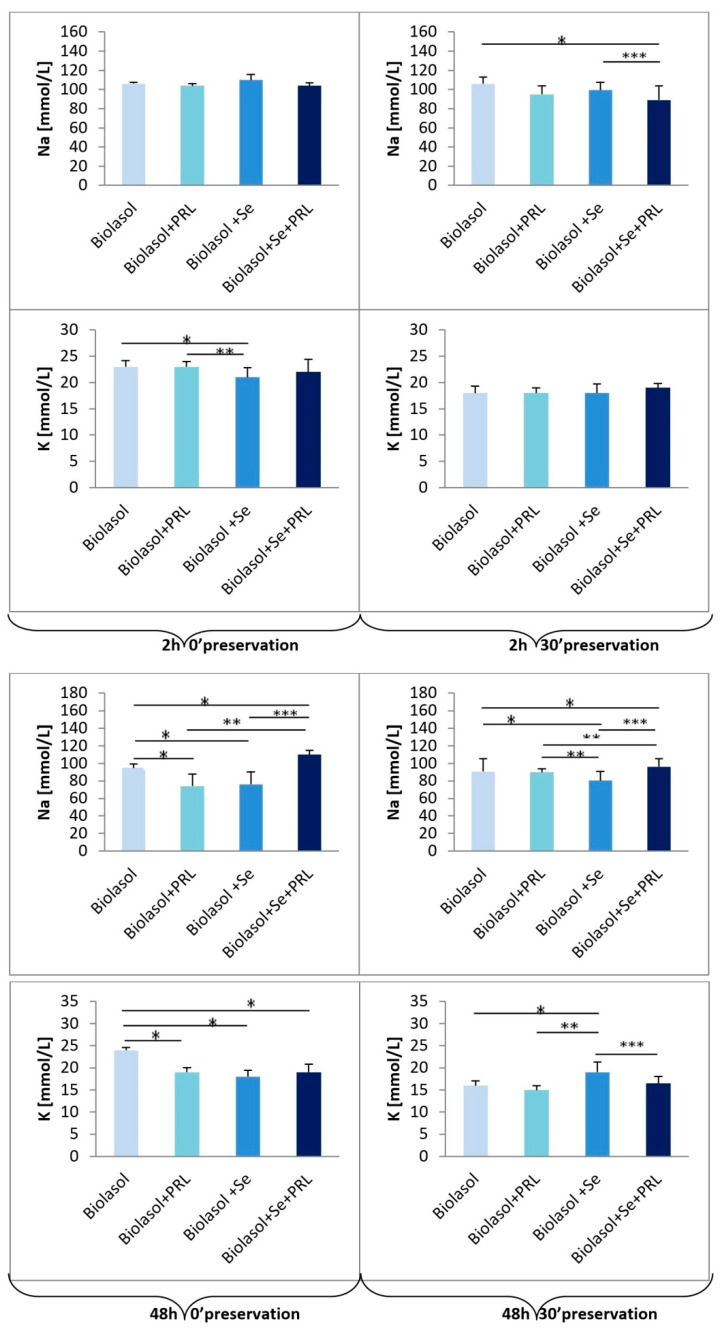
The effect of selenium on sodium and potassium levels in porcine kidneys (2 h 0′; 2 h 30′ and 48 h 0′; 48 h 30′ preservation). Means ± standard deviations (SD) are shown. The symbols represent a significant difference (*p* < 0.05) compared with the Biolasol^®^ group (*), the Biolasol^®^+PRL group (**), and the Biolasol^®^+Se group (***).

The effectiveness of modified Biolasol^®^ in nephron protection was assessed based on the analysis of ALT and AST activities, total protein and urea concentrations as well as Na^+^ and K^+^ levels in the perfundates collected after starting and ending perfusion. A diagnostic tool was used to indirectly assess renal function [22,23,24,25,26,27,28,29]. Analysis of the results obtained confirms the effect of selenium on ischemia-reperfusion injury of isolated porcine kidneys. Figure 2, Figure 3 and Figure 4 present graphs of the effectiveness of storing kidneys in Biolasol^®^ and modified solutions based on markers of their function.

Alanine transaminase and aspartate transaminase activities in the group of kidneys rinsed with Biolasol^®^+Se^4+^+PRL (group B3) were significantly lower compared to the control group (group A), Biolasol^®^+PRL (group B1) and the Biolasol^®^+Se^4+^ group (group B2). In turn, the highest values of these parameters were determined in the perfundates washed with Biolasol^®^+Se^4+^ (group B2). After 2 h 30′, ALT activities were as follows: 18.0 ± 2.1 U/L (group B3) vs. 21.1 ± 4.6 U/L (group A), 19.8 ± 1.6 U/L (group B1), 31.1 ± 5.2 U/L (group B2) (*p <* 0.05), after 48 h 30′, they reached the following values: 6.5 ± 1.9 U/L (group B3) vs. 17.8 ± 1.5 U/L (group A), 14.0 ± 0.4 U/L (group B1), 21.9 ± 1.1 U/L (group B2) (*p <* 0.05). A similar relationship was found in the case of AST activity. The lowest values of this parameter were determined in the perfundates collected after 48 h 30′: 16.6 ± 4.4 U/L (group B3) vs. 20.5 ± 13.7 U/L (group A), 19.6 ± 12.0 U/L (group B1), 54.0 ± 8.6 U/L (group B2) (*p <* 0.05).

Significantly higher protein and urea concentrations were obtained when analysing the perfundates of kidneys washed with Biolasol^®^+Se^4+^ (group B2) compared to the other analysed groups, both after 2.5- and 48.5-h preservation. After 2 h 30′, the protein concentrations were as follows: 1.0 ± 0.2 g/L (group B3) vs. 3.0 ± 1.4 g/L (group A), 2.0 ± 0.2 g/L (group B1), 7.6 ± 1.2 g/L (group B2) (*p <* 0.05). In turn, after 48 h 30′, they reached the following values: 2.1 ± 0.5 g/L (group B3) vs. 5.3 ± 0.7 g/L (group A), 3.0 ± 1.4 g/L (group B1), 6.0 ± 1.0 g/L (group B2) (*p <* 0.05). Urea concentrations after 48.5-h storage compared to the results obtained after 2.5-h storage for each group did not change significantly (group A 2 h 30′ vs group A 48 h 30′; group B1 2 h 30′ vs. group B1 48 h 3p0′; group B2 2 h 30′ vs. group B2 48 h 30′; group B3 2 h 30′ vs. group B3 48 h 30′) (P = NS). However, after 48 h 30′, they reached the following values: 5.9 ± 2.0 mg/dL (group B3) vs. 16.8 ± 1.0 mg/dL (group A), 14.3 ± 1.1 mg/dL (group B1), 22.3 ± 2.2 mg/dL (group B2) (*p <* 0.05).

The observed hyperkalaemia ([K^+^] > 4.9–7.0 mEq/L, the norm for adult pigs) and hyponatraemia ([Na^+^] < 139–152 mEq/L, the norm for adult pigs) result from cellular acidosis due to renal ischemia. The highest K^+^ concentration after 48 h 30′ was found in for the group of kidneys rinsed with Biolasol^®^+Se^4+^ (group B2: 19.0 ± 2.3 mmol/L) vs. Biolasol^®^ (group A: 16.0 ± 1.1 mmol/L), Biolasol^®^+PRL (group B1: 15.0 ± 1.1 mmol/L) and Biolasol^®^+Se^4+^+PRL (group B3: 16.5 ± 1.5 mmol/L) (*p <* 0.05). In turn, the lowest Na^+^ concentration after 48 h 30′ was also observed in the Biolasol^®^+Se^4+^ group (group B2: 80.3 ± 10.4 mmol/L) vs. Biolasol^®^ (group A: 90.7 ± 14.9 mmol/L), Biolasol^®^+PRL (group B1: 90.0 ± 4.3 mmol/L) and Biolasol^®^+Se^4+^+PRL (group B3: 96.3 ± 9.3 mmol/L) (*p <* 0.05).

## 3. Discussion

The introduction of Se^4+^ ions at a dose of 1µg/L into the Biolasol^®^ preservation solution in the form of Na_2_SeO_3_ caused an increase in the activity/concentration of the analysed biochemical parameters: aspartate transaminase, alanine transaminase, urea and protein. This suggests an adverse effect of Se^4+^ on nephron function during ischemia-reperfusion. We suspect that this is due to kidney exposure to selenium (IV), which may have nephrotoxic effects on the tubular epithelium, leading to the loss of normal epithelial function. The normal selenite (SO_3_^2−^) metabolism in the cell is based on its reaction with compounds having sulfhydryl groups (-SH), mainly GSH (reduced glutathione). The product of this reaction is selenodiglutathione (GS-Se-SG), which in the presence of excess GSH is reduced to gluthathioselenol (GSSeH). Selenides, in turn, are selenium donors in the synthesis of selenoproteins [30,31]. It can be supposed that there was a deficiency in reduced glutathione, which is involved in the formation of the bioactive form of selenium, in the isolated porcine kidneys with induced ischemia.

Glutathione reductase is an enzyme found in both the cytosol and mitochondria of the cell. It participates in maintaining the correct concentration of the reduced form of glutathione in the body, catalysing the following reaction: GS-SG+NADPH+H+→2GSH+NADP+ (GS-SG, oxidized glutathione) [32]. Reduced glutathione has strong antioxidant properties. It neutralizes, inter alia, hydrogen peroxide, lipid peroxides, and maintains the appropriate level of -SH groups in proteins. The intracellular glutathione concentration is specific for a particular cell type and amounts to 5–10 mmol/L. GSH is synthesized in the cytoplasm in a reaction involving ATP-dependent enzymes of γ-glutamylcysteinyl synthetase (γSGC) and glutathione synthetase (SG) [33].

The peri-transplantation period is associated with ischemia of the organ during its collection and storage. There occur pathophysiological disorders in the graft that induce inflammation. The resulting ischemia-reperfusion injury contributes to the delayed graft function and/or its acute failure. Hypothermic storage of kidneys in a preservation solution reduces the rate of metabolism and oxygen consumption, which improves their biochemical functions during this period. However, ischemia causes the depletion of intracellular reserves of high-energy ATP compounds and the transition to anaerobic metabolism. Renal reperfusion, in turn, induces increased cellular ROS production and oxidative stress (reperfusion oxygen paradox). Proximal tubule epithelial cells are particularly susceptible to the harmful effects of oxygen free radicals [34]. This is due to the weakening of the antioxidant defence system in the kidneys, mainly because of decreased levels of reduced glutathione (GSH) and GSH:GS-SG as well as superoxide dismutase (SOD) and vitamin E. Generation of oxygen free radicals depletes GSH resources, disrupting the intracellular oxidoreductive balance [35]. Deactivation of glutathione reductase and a reduction in GSH concentration in isolated porcine kidneys are highly probable and may contribute to the disruption of the metabolism of selenite contained in Biolasol^®^.

**Figure 5:**
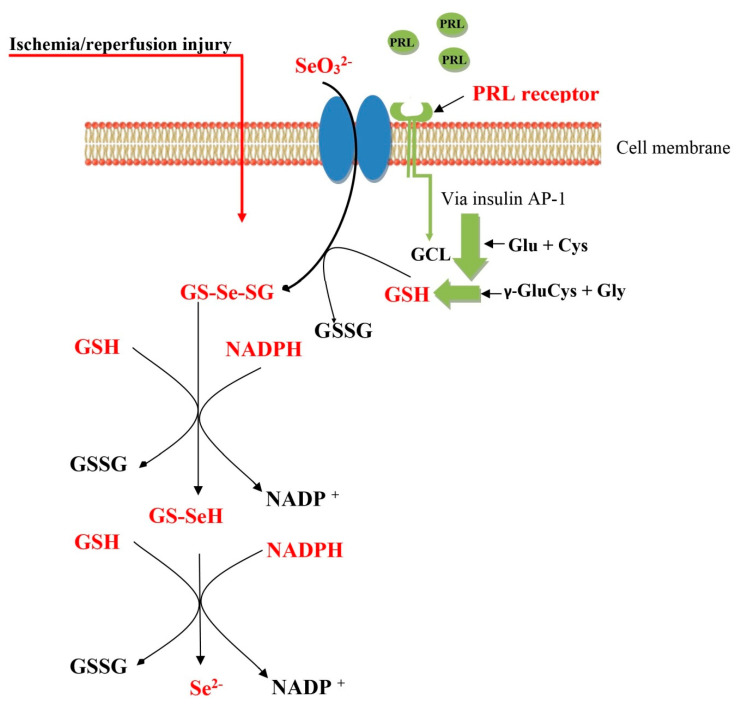
Proposition of the mechanism of prolactin action in the metabolic reduction of selenite (SO_3_^2−^) during ischemia/reperfusion. GSH, reduced glutathione; GSSG, oxidized glutathione; GS-Se-SG, selenodiglutathione; GS-SeH, glutathioselenol; Se^2−^, selenide; PRL, prolactin; GCL, glutamate cysteine ligase; Glu, Glutamate; Cys, Cysteine; Gly, Glycine; γ-GluCys, γ-Glutamyl-cysteine; AP-1, activator protein-1; NADPH, nicotinamide adenine dinucleotide phosphate; NADP+, oxidized form of the electron donor nicotinamide adenine dinucleotide phosphate.

Biolasol^®^ intended, among other uses, for kidney perfusion and preservation was used in the study. Biochemical and histological tests confirm its high effectiveness in protecting nephrons. Biolasol^®^ minimizes ischemia-reperfusion injury, ensures the maintenance of normal homeostasis, reduces tissue swelling and affects the proper maintenance of structural and functional graft integrity [25]. The analysis of the results obtained in this study suggests that Biolasol^®^ modified with the addition of selenite (SO_3_^2−^) and prolactin provides optimal nephron protection. Prolactin is a 23 kDa polypeptide hormone secreted mainly by the eosinophils of the anterior pituitary gland. It is believed that it may act as a pleiotropic cytokine with multidirectional effects on tissues [36]. The presence of prolactin receptors was revealed in the epithelial cells of the proximal tubule and in the parietal epithelial cells of Bowman’s capsule in the kidney [37,38]. Prolactin has anti-apoptotic properties and protects cells against inflammation, showing antioxidant activity [22,23,39,40]. It is suggested that PRL action as an antioxidant results from strengthening the endogenous defence mechanisms of other antioxidants [39]. Prolactin probably does not affect the expression of genes encoding SOD, CAT and GPX [41]. However, it has been found that it may increase the concentration of reduced glutathione (GSH) by two potential mechanisms of action [42]. It may regulate GSH biosynthesis or promote an increase in the concentration of NADPH generation that participates in the reduction of GSSG to GSH [43]. The literature shows that prolactin can modulate the expression and activity of NADPH-producing enzymes, which affects GSH levels. The enzymes responsible for maintaining the proper concentration of NADPH include: isocitrate dehydrogenase, G6PD (glucose 6-phosphate dehydrogenase), PGD (6-phosphogluconate dehydrogenase), NADP malic enzyme [39]. Thébault believes that PRL is a GCL (glutamate cysteine ligase) regulator, an enzyme conditioning GSH synthesis [39]. Therefore, we think that PRL contained in Biolasol^®^ affects the increase in GSH concentration in the cells, which is necessary for the reduction of Se^4+^ to Se^2−^ (selenide). The proposition of the mechanism of prolactin action in the metabolic reduction of selenite (SO32-) is presented in Figure 5 [5,44,45,46]. However, it is necessary to conduct molecular studies in this area.

After 48-h renal storage, we found that both ALT and AST activities determined in Biolasol^®^+Se^4+^+PRL perfusates were by 70% lower than those measured in Biolasol^®^+Se^4+^ perfusates. The system: Se^4+^+PRL affects the integrity of mitochondrial and cytoplasmic membranes [47]. The combination of selenium and prolactin also resulted in lower protein and urea levels (renal dysfunction markers) in the perfusates, by 65% and 74%, respectively. It also minimized changes in transmembrane K^+^ and Na^+^ transport.

An imbalance between the content of extracellular and intracellular potassium affects the regulation of cell volume and the activity of enzymes, including sodium-potassium-ATPase. Ischemia-induced acidosis is the main factor causing disturbed sodium-potassium pump activity. Potassium flows from the cells for exchange with a hydrogen ion causing an increase in K^+^ concentration in the extracellular fluid.

Treśka et al. evaluated the impact of adding selenium to HTK solution on the effectiveness of isolated kidneys of male piglets (the type of selenium compound used was not determined). They have found that selenium minimizes ischemia-reperfusion injury (Table 1). Malondialdehyde concentration decreased with a simultaneous increase in anti-oxidation capacity of plasma [48,49].

Darago et al., in turn, observed that long-term selenium supplementation of male Wistar rats at a dose of 2.8 μg Se (sodium selenite)/ kg body weight did not disturb the homeostasis of this bioelement in the kidneys. There was also no increase in oxidative stress based on GSH and MDA analysis [50]. Therefore, it can be assumed that Biolasol modified with selenium and prolactin is effective in protecting nephrons and is safe. The results obtained are promising and suggest continuing research to optimize the composition of preservation solutions with selenium.

## 4. Materials and Methods

### 4.1. Chemicals

Biolasol^®^ was from FZNP Biochefa (Sosnowiec, Poland) and included: potassium chloride (10 mmol/L), sodium tricitrate (30 mmol/L), sodium hydrocarbonate (5 mmol/L), magnesium fumarate (5 mmol/L), dextran 70 kDa (0.7 g/L), glucose (167 mmol/L), EDTA (5 mmol/L), and ascorbic acid (0.5 mmol/L). The osmotic pressure of the solution is 330 mOsm/L, pH = 7.4. Sodium selenite (Na_2_SO_3_) was from Sigma-Aldrich (Saint Louis, MO, USA. Pig prolactin was from FZNP Biochefa. All substances used in the study were of analytical grade.

### 4.2. Animals

The pigs used for the study came from the Meat Plant H.A.M in Radzionkow, Poland. The study was conducted with the consent of the II Local Ethics Commission for Animal Experiments in Cracow, Poland (number 1046/2013) and in accordance with the European Union Directive (EU guideline 93/119/EC) on the protection of animals during slaughter or killing. 40 kidneys were collected from 20 Polish Large White pigs. Age of animals: 175–180 days, weight: 90–110 kg. The collected kidneys were distributed randomly to the following study groups.

### 4.3. Experimental Groups

The kidneys were divided into four study groups (one control and three experimental groups):

Group A (Biolasol^®^ solution/control; n = 10 kidney). The kidneys were rinsed and stored in Biolasol.

Group B1 (Biolasol^®^ solution with PRL (0.1 µg/L); n = 10 kidney). The kidneys were rinsed and stored in modified Biolasol^®^.

Group B2 (Biolasol^®^ solution with Se^4+^ (1 µg/L); n = 10 kidney). The kidneys were rinsed and stored in modified Biolasol^®^.

Group B3 (Biolasol^®^ solution with Se^4+^ (1 µg/L) and PRL (0.1 µg/L); n = 10 kidney). The kidneys were rinsed and stored in modified Biolasol^®^.

### 4.4. Surgical Procedure

The animals were sacrificed by electrocution (220 V). The kidneys were dissected from the surrounding tissues. The ureter, artery and renal vein were then cut and closed. The renal artery was cannulated using a 40 cm Nelaton CH08 catheter (ConvaTec, Deeside, UK).

### 4.5. Perfusion and Preservation Technique

Immediately after collection, the kidneys were cannulated and placed in a polyethylene bag, filled with 500 mL of the appropriate preservation solution (Group A/B1/B2/B3) at a temperature of 4 °C. The whole was secured with double packaging of sterile bags and placed in an isothermal container. The grafts were stored by simple hypothermia for 2 h. The kidneys were then rinsed at 73.5 mmHg H_2_O providing a continuous flow of the solution. Perfusate samples were collected for biochemistry tests from renal veins at two time points: 0 and 30 min of perfusion. After 30 min, the kidneys were cooled and placed back into a sterile bag filled with 500 mL of the appropriate preservation solution for 48 h. After this time, the renal perfusion scheme and perfusate sampling for biochemistry tests were repeated (at 0 and 30 min of perfusion).

### 4.6. Instruments

The measurements were carried out using a Marcel S330 spectrophotometer (Marcel, Zielonka, Poland). The photometric accuracy of the spectrophotometer was ±0.005 Abs.

### 4.7. Biochemical Determinants

**Figure 6:**
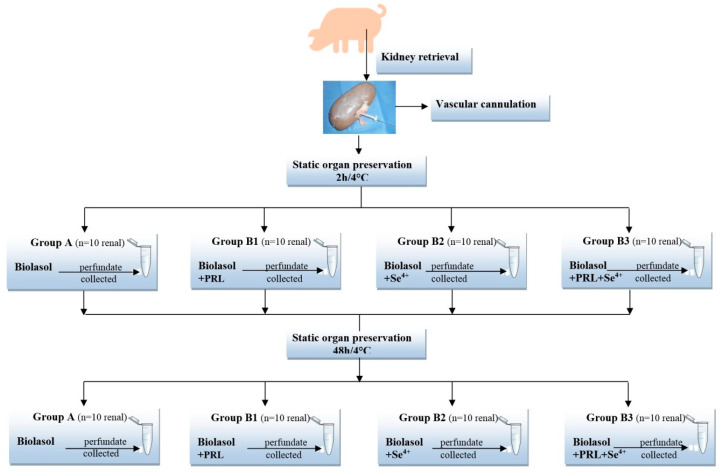
Study design.

The activities of AST (aspartate transaminase) and ALT (alanine transaminase), urea and total protein concentrations (using reagent kits from bioMérieux, Lyon, France) as well as sodium and potassium levels (using Pointe Scientific kits, Marseille, France) were determined in the perfusate samples. The analyses were carried out in accordance with the manufacturers’ instructions. The study design is presented in Figure 6.

### 4.8. Statistical Analysis

The test results are shown as mean ± standard deviation (M ± SD). Comparisons between the groups were made by one-way ANOVA followed by Bonferroni multiple comparison post hoc test (n = 10 for each group). The Statistica version 13.1 software (StatSoft, Kraków Poland) was used. The level of significance was established at *p <* 0.05.

## 5. Conclusions

The obtained results suggest that selenium (IV) as a component of Biolasol^®^ adversely affects the protection of nephrons during the ischemia-reperfusion period. The best graft protection was obtained by using Biolasol^®^ modified with the addition of selenium (IV) at a dose of 1 µg/L and prolactin at a concentration of 0.1 µg/L.

## Figures and Tables

**Table 1:** Effect of the addition of selenium on the effectiveness of the preservation solution.

Author, Year of Publication	Species	Preservation Solution Modification/Cold Ischemia	Outcome Measures, (Intervention, I/control, C)	Selenium Dose	Effects of Selenium
Treśka et al. 2003 [48]	Piglets	HTK /24 h, 4 °C/SCS	I: HTK + Se C: HTK	200 µg	Decreased level of MDA Reduced the production of FOR Higher levels of AOC
Treśka et al. 2003 [49]	Piglets	HTK /24 h, 4 °C/SCS	I: HTK + Se C:HTK	200 µg	Decreased level of MDA Reduced the production of FOR Higher levels of AOC Decreased the intensity of IRS

Abbreviations: MDA: Malondialdehyde; HTK: histidine-tryptophan-ketoglutarate solution, FOR: free oxygen radicals (oxygen superoxide, hydroxyl radical, hydrogen peroxide, and nitric oxide); AOC, anti-oxidation capacity of plasma; IRS, ischemia-reperfusion syndrome.

## References

[1] Mehdi Y., Hornick J.L., Istasse L., Dufrasne I. (2013). Selenium in the environment, metabolism and involvement in body functions. Molecules.

[2] Dominiak A., Wilkaniec A., Wroczyński P., Adamczyk A. (2016). Selenium in the therapy of neurological diseases. Where is it going?. Curr. Neuropharmacol..

[3] Kieliszek M. (2019). Selenium fascinating microelement, properties and sources in food. Molecules.

[4] Jin W., Yoon C., Johnston T.V., Ku S., Ji G.E. (2018). Production of selenomethionine-enriched bifidobacterium bifidum BGN4 via sodium selenite biocatalysis. Molecules.

[5] Guillin O., Vindry C., Ohlmann T., Chavatte L. (2019). Selenium, selenoproteins and viral infection. Nutrients.

[6] Cao L., Zhang L., Zeng H., Wu R.T., Wu T.-L., Cheng W.-H. (2017). Analyses of selenotranscriptomes and selenium concentrations in response to dietary selenium deficiency and age reveal common and distinct patterns by tissue and sex in telomere-dysfunctional mice. J. Nutr..

[7] Zoidis E., Seremelis I., Kontopoulos N., Danezis G. (2018). Selenium-dependent antioxidant enzymes: Actions and properties of selenoproteins. Antioxidants.

[8] Sher L. (2001). Role of thyroid hormones in the effects of selenium on mood, behavior, and cognitive function. Med. Hypotheses.

[9] Youn H.-S., Lim H.J., Choi Y.J., Lee J.Y., Lee M.-Y., Ryu J.-H. (2008). Selenium suppresses the activation of transcription factor NF-κB and IRF3 induced by TLR3 or TLR4 agonists. Int. Immunopharmacol..

[10] Hwang J.-T., Kim Y.M., Surh Y.-J., Baik H.W., Lee S.K., Ha J., Park O.J. (2006). Selenium regulates cyclooxygenase-2 and extracellular signal-regulated kinase signaling pathways by activating AMP-activated protein kinase in colon cancer cells. Cancer Res..

[11] Ruberte A.C., Sanmartín C., Aydillo C., Sharma A.K., Plano D. (2019). Development and therapeutic potential of selenazo compounds. J. Med. Chem..

[12] Duncan A., Talwar D., McMillan D.C., Stefanowicz F., O’Reilly D.S.J., O’Reilly D.S.J. (2011). Quantitative data on the magnitude of the systemic inflammatory response and its effect on micronutrient status based on plasma measurements. Am. J. Clin. Nutr..

[13] Sakr Y., Reinhart K., Bloos F., Marx G., Russwurm S., Bauer M., Brunkhorst F.M. (2007). Time course and relationship between plasma selenium concentrations, systemic inflammatory response, sepsis, and multiorgan failure. Br. J. Anaesth..

[14] Toufektsian M.C., Boucher F., Pucheu S., Tanguy S., Ribuot C., Sanou D., Tresallet N., de Leiris J. (2000). Effects of selenium deficiency on the response of cardiac tissue to ischemia and reperfusion. Toxicology.

[15] Poltronieri R., Cevese A., Sbarbati A. (1992). Protective effect of selenium in cardiac ischemia and reperfusion. Cardioscience.

[16] Tanguy S., Boucher F., Besse S., Ducros V., Favier A., De Leiris J. (1998). Trace elements and cardioprotection: Increasing endogenous glutathione peroxidase activity by oral selenium supplementation in rats limits reperfusion-induced arrhythmias. J. Trace Elements Med. Boil..

[17] Zapletal C., Heyne S., Breitkreutz R., Gebhard M.-M., Golling M. (2008). The influence of selenium substitution on microcirculation and glutathione metabolism after warm liver ischemia/reperfusion in a rat model. Microvasc. Res..

[18] Hasanvand A., Abbaszadeh A., Darabi S., Nazari A., Gholami M., Kharazmkia A. (2016). Evaluation of selenium on kidney function following ischemic injury in rats; protective effects and antioxidant activity. J. Ren. Inj. Prev..

[19] Ahmadvand H., Babaeenezhad E., Nayeri H., Nezhad Z.Z. (2018). Selenium effects on antioxidant and inflammatory indices in renal ischemia-reperfusion injury in rats. J. Ren. Inj. Prev..

[20] Kim H.-Y. (2013). The methionine sulfoxide reduction system: Selenium utilization and methionine sulfoxide reductase enzymes and their functions. Antioxidants Redox Signal..

[21] Qazi I.H., Angel C., Yang H., Zoidis E., Pan B., Wu Z., Ming Z., Zeng C., Meng Q., Han H.-B. (2019). Role of selenium and selenoproteins in male reproductive function: A review of past and present evidences. Antioxidants.

[22] Ryszka F., Dolińska B., Czyż K., Jelińska M., Strabel A., Bocheńska J. (2016). Effect of recombinant human prolactin addition to biolasol solution on biochemical indicators in perfundates of porcine kidneys. Transplant. Proc..

[23] Ostróżka-Cieślik A., Dolińska B., Ryszka F. (2018). The effect of modified biolasol solution on the efficacy of storing isolated porcine kidneys. BioMed Res. Int..

[24] Ostróżka-Cieślik A., Dolińska B. (2020). The Role of hormones and trophic factors as components of preservation solutions in protection of renal function before transplantation: A review of the literature. Molecules.

[25] Cierpka L., Ryszka F., Dolińska B., Smorąg Z., Słomski R., Wiaderkiewicz R., Caban A., Budziński G., Oczkowicz G., Wieczorek J. (2014). Biolasol: Novel perfusion and preservation solution for kidneys. Transplant. Proc..

[26] Ryszka F., Dolińska B., Ostróżka-Cieślik A., Caban A., Cierpka L. (2012). Comparing the effect of Biolasol^®^ and HTK solutions on maintaining proper homeostasis, indicating the kidney storage efficiency prior to transplantation. Ann. Transplant..

[27] Almalki A.M., Ajarem J., Altoom N., Al-Otaibi F.S., Maodaa S.N., Allam A.A., Mahmoud A.M. (2019). Effects of mining activities on *Gerbillus nanus* in Saudi Arabia: A biochemical and histological study. Animals.

[28] Uslu G.A., Uslu H., Adalı Y. (2019). Hepatoprotective and nephroprotective effects of *Trigonella foenum*-*graecum L. (Fenugreek*) seed extract against sodium nitrite toxicity in rats. Biomed. Res. Ther..

[29] Ostróżka-Cieślik A., Dolińska B., Ryszka F. (2020). Effect of lutropin concentration on the efficiency of isolated porcine kidney storage in modified biolasol solution. Transplant. Proc..

[30] Siwek B., Bahbouth E., Serra M., Sabbioni E., De Pauw-Gillet M.-C., Bassleer R. (1994). Effect of selenium compounds on murine B16 melanoma cells and pigmented cloned pB16 cells. Arch. Toxicol..

[31] Björnstedt M., Kumar S., Holmgren A. (1992). Selenodiglutathione is a highly efficient oxidant of reduced thioredoxin and a substrate for mammalian thioredoxin reductase. J. Boil. Chem..

[32] Twardoch M., Lodwich M., Mazur B. (2016). Allergy and oxidative stress. Ann. Acad. Med. Silesiensis.

[33] Meister A. (1983). Selective modification of glutathione metabolism. Science.

[34] Chien C.T., Lee P.H., Chen C.F., Ma M.C., Lai M.K., Hsu S.M. (2001). De novo demonstration and co-localization of free-radical production and apoptosis formation in rat kidney subjected to ischemia/reperfusion. J. Am. Soc. Nephrol..

[35] Shang Y., Siow Y.L., Isaak C.K. (2016). Downregulation of glutathione biosynthesis contributes to oxidative stress and liver dysfunction in acute kidney injury. Oxidative Med. Cell. Longev..

[36] Grattan D.R. (2016). The eyes have it! Protective role of prolactin in the retina. EBioMedicine.

[37] Mountjoy K., Cowden E.A., Dobbie J.W., Ratcliffe J.G. (1980). Prolactin receptors in the rat kidney. J. Endocrinol..

[38] Sakai Y., Hiraoka Y., Ogawa M., Takeuchi Y., Aiso S. (1999). The prolactin gene is expressed in the mouse kidney. Kidney Int..

[39] Thébault S. (2017). Potential mechanisms behind the antioxidant actions of prolactin in the retina. Exp. Eye Res..

[40] Goffin V., Touraine P. (2015). The prolactin receptor as a therapeutic target in human diseases: Browsing new potential indications. Expert Opin. Ther. Targets.

[41] García R.M., Zamarripa D.A., Arnold E., Ruiz-Herrera X., Imm R.N., Cruz G.B., Adan N., Binart N., Riesgo-Escovar J., Goffin V. (2016). Prolactin protects retinal pigment epithelium by inhibiting sirtuin 2-dependent cell death. EBioMedicine.

[42] Masella R., Di Benedetto R., Varì R., Filesi C., Giovannini C. (2005). Novel mechanisms of natural antioxidant compounds in biological systems: Involvement of glutathione and glutathione-related enzymes. J. Nutr. Biochem..

[43] Lu S.C. (2009). Regulation of glutathione synthesis. Mol. Asp. Med..

[44] Herrero E., Wellinger R.E. (2015). Yeast as a model system to study metabolic impact of selenium compounds. Microb. Cell.

[45] Letavayová L., Vlčková V., Brozmanová J. (2006). Selenium: From cancer prevention to DNA damage. Toxicology.

[46] Sun H., Rathinasabapathi B., Wu B., Luo J., Pu L.-P., Ma L.Q. (2014). Arsenic and selenium toxicity and their interactive effects in humans. Environ. Int..

[47] Lameire N., Van Biesen W., Vanholder R. (2005). Acute renal failure. Lancet.

[48] Třeška V., Kuntscher V., Molacek J., Kobr J., Racek J., Trefil L. (2003). Can the ischemia-reperfusion syndrome in transplanted kidneys procured from non-heart-beating donors be influenced by adding selenium into the reperfusion solution? An experimental study. Transplant. Proc..

[49] Třeška V., Kuntscher V., Molacek J., Kobr J., Racek J., Trefil L. (2003). Can ischemia-reperfusion syndrome in transplanted kidneys procured from non-heart-beating donors be influenced by adding selenium into the reperfusion solution? An experimental study. Transplant. Proc..

[50] Darago A., Sapota A., Nasiadek M., Klimczak M., Bruchajzer E., Kilanowicz A. (2017). The influence of subchronic zinc and/or selenium supplementation in Wistar rats on homeostasis of these bioelements in the Sidney. Bromat. Chem. Toksykol..

